# Heredity
*"Heredity: a Study, with special reference to Disease." By R. A. Douglas Lithgow, LL.D., F.S.A., M.R.C.P., L.R.C.S. Edin., L.S.A. Lond., &c. London: Bailliere, Tindall, and Cox, 20, King William-street, Strand.


**Published:** 1889-05-25

**Authors:** 


### HEREDITY.*

IF doctors had time to preach sermons, there can be little doubt that they would insist strenuously on the wider recognition of the fundamental doctrine that underlies and directs all evolution, human, and otherwise, the law of heredity. They would teach that "the sins of the fathers are visited on the children," not in the angry vindictiveness of an irresponsible deity, but in the inevitable process of the law of generation. That law is, to a great extent, a mystery. The most unimaginative physiologist must sometimes be struck with wonder when he reflects that in the embryonic vesicle, formed by the union of two simple cells, lies *in posse* all the powers and passions, the merits and defects, the beauty or deformity of the parents. In no human being do we ever see the perfect combination of the qualities of both father and mother; the influence of one or other always preponderates, but that of the other is latent, as may be inferred from the fact that at one period of life a child may resemble its father, while at another the mother's characteristics come to the front. The "family likeness" of which we speak so lightly, extending as it does so far beyond form and feature, is one of the greatest mysteries in creation, and makes anyone who reflects on the subject feel at once an awful helplessness in face of the frequent contest of inherited tendencies that goes on in every fibre of his being, and an intense determination to guide these tendencies in such a fashion that it shall not be the worst of them that will descend unimpaired or strengthened to unborn generations. To use the language of the old theologians, who often perceived a truth even where they were most rash in expression, in heredity the doctrines of predestination and freewill are combined. A man inherits from father and mother a legacy of qualities, good and evil, of inherited tendencies to certain diseases and immunity from others, as surely as he inherits their money or their debts; with this difference, that no human power can set aside or alter the bequest. Only he himself can choose, and by his will decide—but then strength or weakness of will is itself an inheritance—which qualities he will strive to dissipate and which to put out to usury.

Heredity may be of many kinds. The commonest is direct heredity, in which qualities pass from father to son, or, more frequently in a diagonal line, from father to daughter, and mother to son. Of course, the transmission is rarely complete. A girl may have her mother's face and her father's temper, or *vice versâ*. The combinations and variations are as endless as those of a kaleidoscope, and yet in all a marked type will predominate, which sets the family apart from every other. Atavism, or reversion to an ancestral type, is by no means uncommon, and in close connection with it is indirect heredity, in which a child resembles an uncle or cousin, or some other relative out of the line of direct descent. This is, in fact, only a reversion to some ancestral type common to both, and often too remote to be recognised.

In families that have attained distinction of one kind or another, we can note the permanence of certain characteristics. The Guises were bold, rash, and proud, yet marked by a certain charm of manner that won them power over men, and often gained forgiveness for their crimes. These qualities they not only transmitted in their own line, but passed on, through Mary of Lorraine, to the faithless, impetuous, fascinating Stuarts, who, like the Bourbons, "learned nothing and forgot nothing" through defeat and adversity. In other departments we see the influence of heredity in such families of statesmen as the Medici, the Pitts, and the Peels. In science, no more notable example could be found than the Darwins, while in music the Bachs, and in art the three generations of Vernets are pre-eminent.

Among physical characteristics may be mentioned the numerous diseases that "run in families." A hereditary predisposition to gout is supposed to be rather respectable than otherwise, presumably as implying wealth to spend on good living, though when the diathesis is firmly established in a family, it will come out in circumstances of poverty and hardship. Other diseases are equally hereditary. Phthisis, our specially English disease, often carries off whole families. In other ailments, such as epilepsy and insanity, only one member of a family may inherit the taint—may become, as it were, the scapegoat of the rest. In all these, the predisposition may miss a generation, or even two; but will recur with unfailing certainty at last. Sex also affects heredity. Men are more liable to acute inflammatory attacks than women, who, on the other hand, are specially subject to nervous affections. The same constitution which causes a man to be cut off in the prime of life may allow his daughter to reach old age, subject only to a certain lack of tone. She will be one of those "creaking doors that hang long on the hinge," unless some accident brings an extra burden to be borne by the particular organ that inherits the weakness.

With careful treatment the health may improve, the hereditary predisposition be lessened. For, as a rule, it is only a predisposition that is inherited, though children have been born with heart or liver disease already established, and if the mothers have suffered from any epidemic disease, such as scarlet-fever or smallpox, the infants are affected by it. Even stranger cases have been known, where the disease has passed the mother, and fixed on the unborn child. Such instances are not exact examples of heredity, except in as far as they show that a parent, without being personally affected, may transmit disease to offspring.

Where, however, only a tendency, not an established disease, has been inherited, careful choice of occupation and residence may do much to eradicate it. We recall the case of two brothers, in whom a hereditary tendency to tubercular disease appeared in the form of atavism. They themselves were free from it, but their mother had died of consumption, and the taint appeared again in the children of each. After losing one child, the elder brother, wise in time, removed his whole family to Australia, where their health has steadily improved. The other remained at home, and now, of seven children, only one daughter survives, and her health is a constant cause of anxiety. The diseases which have carried off the others have not all been the same, but they may all be traced to the tubercular diathesis, modified to different forms by age, sex, and circumstances of life.

It is indeed rather rash to say that any one disease is strictly hereditary. It is rather the predisposition than the ailment that is inherited, and this comes out in different forms, according as the individual is exposed to infection in youth, manhood, or maturer age. In many cases the disease may take a mental form in one member of a family and a physical form in another. It is true, of course, that insanity is usually a result of some physical lesion, and may even be cured by means of cranial surgery; but it is convenient to define as mental disease that in which the only symptom is some imperfection in the functions of the brain. Thus one individual may be mad, while his sister, though perfectly sane, may suffer from cancer or scrofula. This may result from two morbid taints being inherited from different ancestors, or it may be the same taint taking different forms under different circumstances.

It is often asked if cancer is hereditary. On this point

* "Heredity: a Study, with special reference to Disease." By R. A. Douglas Lithgow, LL.D., F.S.A., M.R.C.P., L.R.C.S. Edin., L.S.A. Lond., &c. London: Bailliere, Tindall, and Cox, 20, King William-street, Strand.

authorities differ greatly. The general belief is that only a general taint is inherited, which may remain in abeyance or may be developed by circumstances; but when one hears of a case like that recorded by Dr. Green, of a lady who was the fifth of her family to suffer from cancer within two generations, one cannot but think that the disease itself is derived from an ancestor. Instances such as these could be quoted in number from Dr. Lithgow's interesting book, on which our remarks are largely founded.

On the side of morals heredity is equally marked. Indeed, it cannot but raise in our minds a question as to the absolute responsibility of any human being. The inherited vices of our ancestors may conquer the aspirations of our better nature. On the other hand, a legacy of pure instincts may save us from the fires of temptation. In face of this we may punish, but we dare not reprobate, our erring brother. We have not fallen into the same pit as he; but God alone can judge if, according to our circumstances and opportunities, we have not erred as grievously, and we must needs remind ourselves of the plea of the poet:

"Then at the balance let's be mute,
We never can adjust it;
What's done we partly may compute,
We know not what's resisted."

